# Benzo[*a*]pyrene diol epoxide suppresses retinoic acid receptor-β_2 _expression by recruiting DNA (cytosine-5-)-methyltransferase 3A

**DOI:** 10.1186/1476-4598-9-93

**Published:** 2010-04-28

**Authors:** Fei Ye, Xiao-Chun Xu

**Affiliations:** 1 Department of Clinical Cancer Prevention, Unit 1360, The University of Texas M. D. Anderson Cancer Center, 1515 Holcombe Boulevard, Houston, TX 77030, USA

## Abstract

Tobacco smoke is an important risk factor for various human cancers, including esophageal cancer. How benzo [*a*]pyrene diol epoxide (BPDE), a carcinogen present in tobacco smoke as well as in environmental pollution, induces esophageal carcinogenesis has yet to be defined. In this study, we investigated the molecular mechanism responsible for BPDE-suppressed expression of retinoic acid receptor-beta2 (RAR-β_2_) in esophageal cancer cells. We treated esophageal cancer cells with BPDE before performing methylation-specific polymerase chain reaction (MSP) to find that BPDE induced methylation of the RAR-β_2 _gene promoter. We then performed chromatin immunoprecipitation (ChIP) assays to find that BPDE recruited genes of the methylation machinery into the RAR-β_2 _gene promoter. We found that BPDE recruited DNA (cytosine-5-)-methyltransferase 3 alpha (DNMT3A), but not beta (DNMT3B), in a time-dependent manner to methylate the RAR-β_2 _gene promoter, which we confirmed by reverse transcription-polymerase chain reaction (RT-PCR) analysis of the reduced RAR-β_2 _expression in these BPDE-treated esophageal cancer cell lines. However, BPDE did not significantly change DNMT3A expression, but it slightly reduced DNMT3B expression. DNA methylase inhibitor 5-aza-2'-deoxycytidine (5-Aza) and DNMT3A small hairpin RNA (shRNA) vector antagonized the effects of BPDE on RAR-β_2 _expressions. Transient transfection of the DNMT3A shRNA vector also antagonized BPDE's effects on expression of RAR-β_2_, c-Jun, phosphorylated extracellular signal-regulated protein kinases 1/2 (ERK1/2), and cyclooxygenase-2 (COX-2), suggesting a possible therapeutic effect. The results of this study form the link between the esophageal cancer risk factor BPDE and the reduced RAR-β_2 _expression.

## Findings

Tobacco smoke is an important cause of human cancers, as it contains more than 60 carcinogens [1-4], which are major risk factors for cancers of the head and neck, lung, esophagus, pancreas, and bladder [5-9]. Benzo [*a*]pyrene diol epoxide (BPDE), a carcinogen present in tobacco smoke and environmental pollution, has been shown to induce gene mutations (such as in *p53 *and *KRAS *genes) in vitro [10-13]. Previously, we identified and cloned several BPDE-binding genes (such as *ATM *and *BRCA2*) and the cytosine-phosphate-guanine (CpG) islands of various gene promoters [14]. Cigarette smoke has been shown to cause morphologic changes and the loss of retinoic acid receptor-beta2 (RAR-β_2_) expression in the lung tissues of experimental animals [15]. Cigarette smoke, specifically the tobacco carcinogen 4-(methylnitrosamino)-1-(3-pyridyl)-1-butanone, has also been shown to induce the methylation of the RAR-β_2 _gene promoters in murine lung cancer models [16]. We have also previously shown that RAR-β_2 _expression is suppressed in premalignant and malignant esophageal cells [17-19]. Consequently, expression of epidermal growth factor receptor (EGFR), extracellular signal-regulated protein kinases 1/2 (ERK1/2), activated protein-1 (AP-1), and cyclooxygenase-2 (COX-2) are induced by BPDE [19]. Numerous studies have demonstrated that RAR-β_2 _expression is frequently and progressively lost in premalignant and malignant tissues of the head and neck, lung, esophagus, pancreas, mammary gland, prostate, and other sites [20-22]. Lost expression of RAR-β_2 _in these various human cancers has been shown to be due to hypermethylation of its gene promoter [23-27]. However, it is still unknown if, and if so, how BPDE suppresses the expression of RAR-β_2 _and induces methylation of its gene promoter.

**Figure 1:**
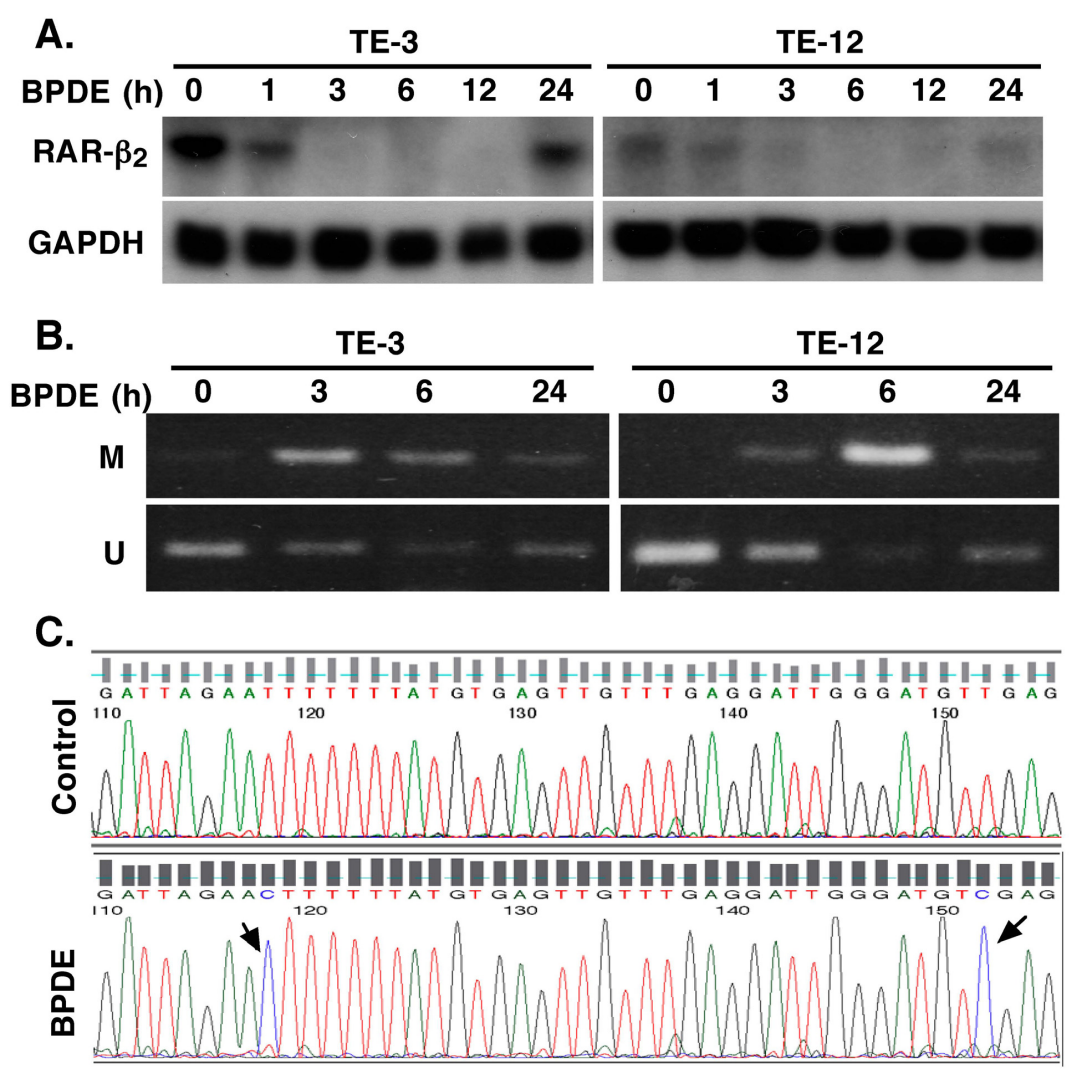
**Suppression of RAR-β_2 _expression by BPDE**. (A) Northern blotting. Esophageal cancer cell lines TE-3 and TE-12 were grown and treated with 1 μM BPDE for up to 24 h. RNA was then isolated from the cells and subjected to Northern blotting analysis of RAR-β_2 _expression. (B) MSP. Esophageal cancer cell lines TE-3 and TE-12 were grown and treated with 1 μM BPDE for up to 24 h. Genomic DNA was then isolated from the cells and subjected to PCR analysis of RAR-β_2 _gene promoter methylation. M, methylated RAR-β_2 _gene promoter; U, unmethylated RAR-β_2 _gene promoter. (C) Sequencing histogram (matching GenBank accession number X56849) of a partial RAR-β_2 _gene promoter. TE-3 cells were treated with or without 1 μM BPDE for 12 h, and genomic DNA was extracted and subjected to MSP analysis. The PCR product was then cloned into a TA cloning vector. The clones carrying RAR-β_2 _gene promoter were then sequenced in our institutional DNA sequencing facility. Compared with the controls, BPDE-treated cells showed two BPDE-methylated sites. Note: Bisulfite converted all C to T, but methylated C cannot be converted, which is the principle of the MSP assay.

**Figure 2:**
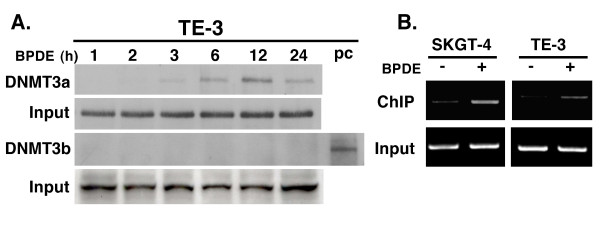
**ChIP assay**. Esophageal cancer TE-3 cells were grown and treated with 1 μM BPDE for up to 24 h. After that, the cells were fixed with formaldehyde for 10 min, and the nuclei were then isolated, sonicated, and immunoprecipitated with anti-BPDE antibodies. (A) Immunoprecipitated protein was subjected to Western blotting analysis of DNMT3A and DNMT3B expression. Protein without anti-BPDE antibody immunoprecipitation (input) was used as the control. PC, positive control. (B) Immunoprecipitated DNA was subjected to PCR analysis of RAR-β_2 _gene promoter.

**Figure 3:**
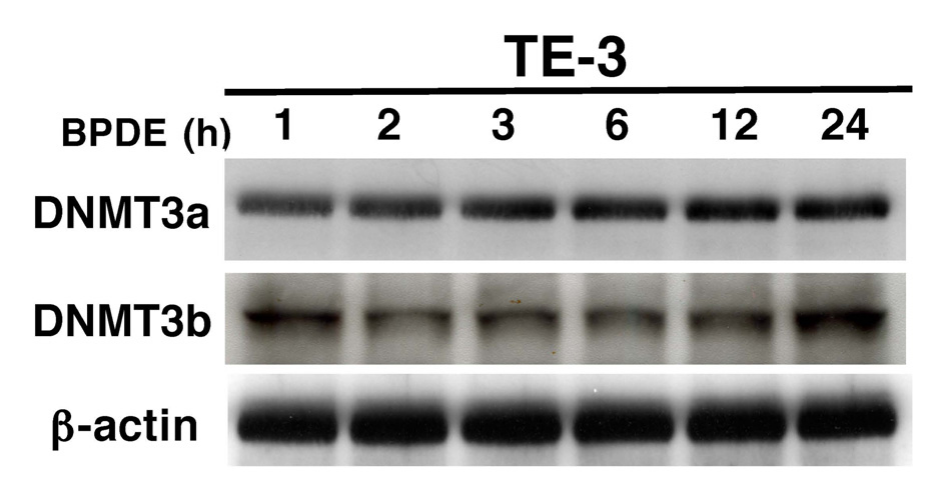
**Effect of BPDE on DNMT3A and DNMT3B expression**. TE-3 cells were grown and treated with 1 μM BPDE for up to 24 h. After that, total cellular protein was extracted and subjected to Western blotting analysis of DNMT3A and DNMT3B expression.

**Figure 4:**
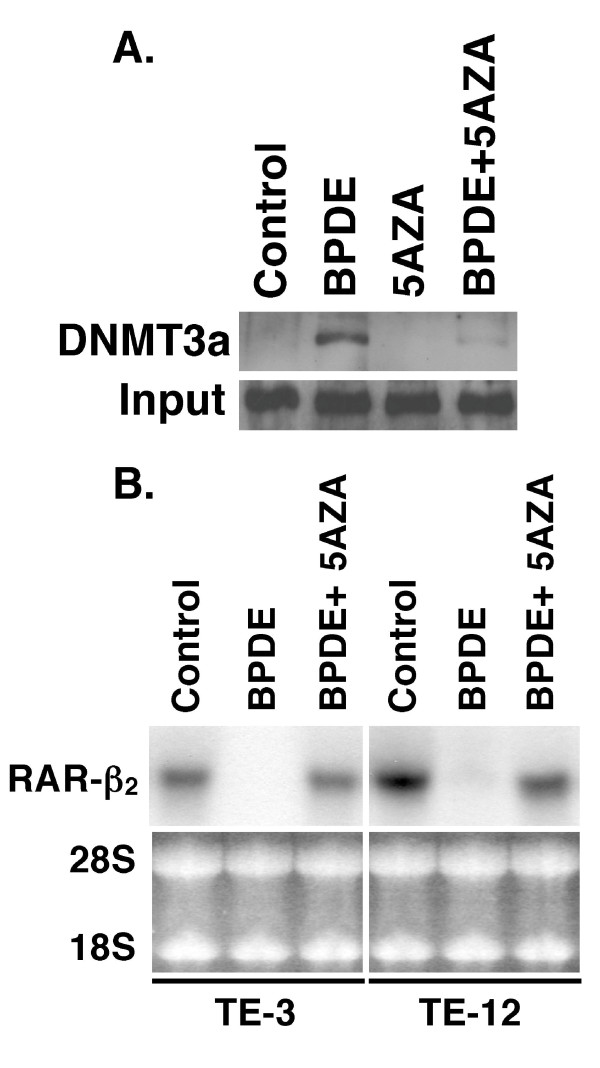
**Reversal of BPDE's effect on RAR-β_2 _expression using 5-Aza**. (A) ChIP assay. Esophageal cancer TE-3 cells were grown and treated with or without 1 μM BPDE, 10 μM 5-Aza, or the combination of both for 12 h, and the cells were then subjected to ChIP analysis with anti-BPDE antibodies. The immunoprecipitated protein was subjected to Western blotting analysis of DNMT3A expression. Proteins without anti-BPDE antibody immunoprecipitation (input) were used as controls. (B) Northern blotting. Total RNA from TE-3 and TE-12 cells with the same treatment was isolated and subjected to Northern blotting analysis of RAR-β_2 _expression.

**Figure 5:**
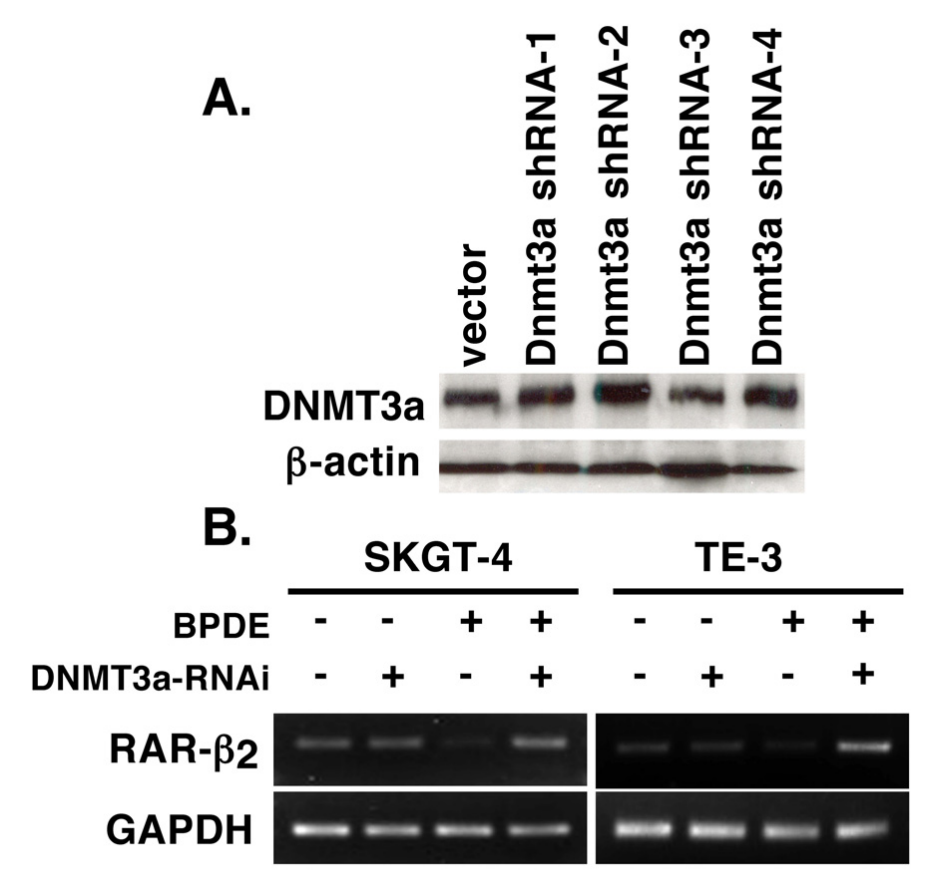
**Effects of DNMT3A shRNA**. (A) Knockdown of DNMT3A expression by DNMT3A shRNA. Esophageal cancer TE-3 cells were grown and transiently transfected with DNMT3A 29 mer shRNA constructs in pRS vector; 48 h later, total cellular protein was extracted and then subjected to Western blotting analysis of DNMT3A expression. (B) RT-PCR analysis of RAR-β_2 _mRNA expression. Esophageal cancer TE-3 cells were grown and transiently transfected with DNMT3A shRNA-3 vector; 48 h later, the cells were treated with or without 1 μM BPDE for 12 h and RNA was then isolated from the cells and subjected to RT-PCR analysis.

**Figure 6:**
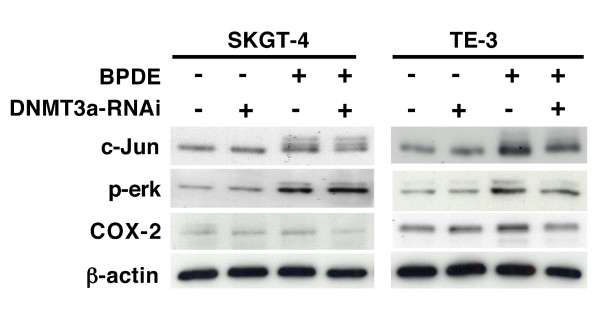
**DNMT3A shRNA antagonized BPDE's effect on gene expression to different levels**. Esophageal cancer SKGT-4 and TE3 cells were grown and transiently transfected with the empty vector or DNMT3A shRNA-3 vector for 48 h. The cells were then treated with or without 1 μM BPDE for 12 h, and the total cellular protein was extracted and subjected to Western blotting analysis of c-Jun, phosphorylated-ERK1/2 and COX-2 expression.

In this study, we first confirmed our previous finding [17,19] that BPDE treatment inhibited RAR-β_2 _expression and after 24 h BPDE treatment, RAR-β_2 _was reexpressed (Figure 1A) and then we showed that BPDE induced the methylation of the RAR-β_2 _gene promoter and in 24 h BPDE treatment, some DNA still remains methylated (Figure 1B). Sequencing data from methylation-specific polymerase chain reaction (MSP) products confirmed thymine (T)-to-cytosine (C) transitions, indicating that BPDE induced methylation of the RAR-β_2 _gene promoter (Figure 1C). The reason for reexpression of RAR-β_2 _after 24 h BPDE treatment may be because of BPDE-activated DNA repair mechanism and in our previous paper, we showed that BPDE treatment induced ATM expression, an early protein in the DNA repair pathway after esophageal cancer cells were insulted by BPDE and that ATM expression was associated with tobacco smoke exposure in esophageal cancer tissues [14,28]. Next, we determined the underlying molecular mechanism responsible for BPDE-induced RAR-β_2 _promoter methylation by performing a chromatin immunoprecipitation (ChIP) assay with anti-BPDE antibodies and then a Western blotting analysis of DNA (cytosine-5-)-methyltransferase 3 alpha (DNMT3A) and beta (DNMT3B) expression and a polymerase chain reaction (PCR) analysis of RAR-β_2 _gene promoter expression. The immunoprecipitated protein showed that BPDE recruited the DNMT3A protein, but not the DNMT3B protein, to RAR-β_2 _gene promoter sites in a time-dependent manner (Figure 2A). The immunoprecipitated DNA confirmed that BPDE and DNMT3A bind to the RAR-β_2 _gene promoter. Although RAR-β_2 _was reexpressed 24 h after BPDE treatment, there were detectable levels of methylated DNA (Figure 1B) that may contribute to weaker DNMT3a-binding to the RAR-β_2 _gene promoter in Figure 2A. However, we found that BPDE did not significantly change the expression levels of the DNMT3A protein, but it did slightly decrease DNMT3B protein levels (Figure 3), which is similar to a previous study showed that 24 h treatment with cigarette smoke significantly downregulated DNMT3B but just slightly induced DNMT3a expression in lung cancer cells [29]. Furthermore, we determined that the DNA methylase inhibitor 5-aza-2'-deoxycytidine (5-Aza) and DNMT3A small hairpin RNA (shRNA) vector antagonized the effects of BPDE on the methylation of the RAR-β_2 _gene promoter. ChIP assay data showed that 5-Aza treatment inhibited the recruitment of DNMT3A by BPDE to the RAR-β_2 _gene promoter site in BPDE-treated TE-12 cells (Figure 4A). Northern blot analysis indeed showed that RAR-β_2 _mRNA expression was restored in TE-12 cells after treatment with 5-Aza (Figure 4B). After these tests, we determined the ability of four DNMT3A shRNA constructs to knock down DNMT3A expression and found that DNMT3A shRNA-3 was able to sufficiently knock down expression levels of the DNMT3A protein (Figure 5A). We therefore performed transient gene transfection experiments followed by reverse transcription-polymerase chain reaction (RT-PCR) analyses and found that the knockdown of DNMT3A expression antagonized the ability of BPDE to suppress RAR-β_2 _expression (Figure 5B). Consequently, we sought to determine if knockdown of DNMT3A could antagonize the effect of BPDE on expression of these genes. We found that transient transfection with DNMT3A shRNA vector indeed antagonized BPDE's inhibition of RAR-β_2 _expression (Figure 5B) and its upregulation of c-Jun, phosphorylated ERK1/2, and COX-2 in BPDE-treated SKGT4 and TE-3 cells (Figure 6).

Our current findings provide further evidence that BPDE may play a role in esophageal cancer development and progression by suppressing RAR-β_2 _expression. As mentioned above, numerous studies have demonstrated that RAR-β_2 _expression is frequently and progressively lost in premalignant and malignant tissues and cells [20-22]. These studies clearly indicate that RAR-β_2 _functions as a tumor suppressor gene. RAR-β_2 _gene promoter methylation is believed to be responsible for the lost expression of RAR-β_2 _in various human cancers, including esophageal cancer [20-27]. Lost expression of RAR-β_2 _and methylation of the RAR-β_2 _gene promoter have been used as diagnostic markers of tumorigenesis [22]. For example, methylation of the RAR-β_2 _gene promoter has been found in early-stage breast cancer [23] and has been detected in the bronchial aspirates of lung cancer patients at a much higher frequency than in patients with benign lung disease [24]. Furthermore, the RAR-β_2 _gene promoter has been detected at a high level of methylation in esophageal cancer and was found to be associated with *RAR-β*_2 _gene silencing in this disease [25]. Cigarette smoke has been shown to downregulate RAR-β_2 _expression, but not that of RAR-α or RAR-γ in the lungs of ferrets [15]. However, the cause of this lost RAR-β_2 _expression or RAR-β_2 _gene promoter methylation is not fully understood.

The current study mechanistically links the esophageal cancer risk factor BPDE to suppressed RAR-β_2 _expression and RAR-β_2 _gene promoter methylation, which may help in the development of novel strategies against this deadly disease by using chemopreventive agents to antagonize the effects of BPDE on esophageal epithelial cells. Recent studies have shown that cigarette smoke, specifically the tobacco carcinogen 4-(methylnitrosamino)-1-(3-pyridyl)-1-butanone, reduced RAR-β_2 _expression or induced RAR-β_2 _gene promoter methylation in experimental animals [16]. Epigallocatechin gallate induced a concentration- and time-dependent reversal of RAR-β_2 _gene promoter methylation in esophageal cancer cell lines, resulting in the restoration of RAR-β_2 _expression [30]. The factors known to be associated with aberrant CpG island methylation include local DNA structure changes, carcinogen exposure, increased DNA-methyltransferase activity, and microsatellite instability [31,32]. Tobacco carcinogens are also known factors in the methylation of various tumor suppressor genes, including the RAR-β_2 _gene promoter [33-35]. However, the defined causes of CpG island methylation in cancer are largely unknown. In the current study, we found that BPDE recruited DNMT3A to methylate the RAR-β_2 _gene promoter and thus silence its gene expression, which in turn, may contribute to the malignant transformation of esophageal epithelial cells. Taken together, the results of this study form the link between the esophageal cancer risk factor BPDE and the reduced RAR-β_2 _expression, which may help in the development of novel strategies against this now deadly disease by antagonizing the effects of BPDE on esophageal epithelial cells with anti-methylation agents.

## Materials and methods

### Cell culture and drug treatment

Esophageal squamous cancer cell lines TE-3 and TE-12 and adenocarcinoma cell line SKGT4 were grown and maintained as described elsewhere [17-19]. BPDE was purchased from Midwest Research Institute (Kansas City, MO) and 5-Aza from Sigma (St. Louis, MO). The treatment schedules were the same as those used in our previous studies [17-19].

### RNA isolation and Northern blotting

TRIzol reagent (Invitrogen, Carlsbad, CA) was used to extract RNA from monolayer cultures, and the plasmid pRC/CMV (Invitrogen, San Diego, CA), which contains human RAR-β_2 _cDNA, was prepared for using as the Northern blotting probe as previously described [36].

### MSP and DNA sequencing

DNA isolated from these cells was subjected to MSP using an MSP kit (Zymed, South San Francisco, CA) according to the manufacturer's instructions. The primers used to amplify the methylated RAR-β_2 _genes were 5'-TCGAGAACGCGAGCGATTCG-3' and 5'-GACCAATCCAACCGAAACGA-3'. The primers used to amplify the unmethylated RAR-β_2 _genes were 5'-TTGAGAATGTGAGTGATTTGA-3' and 5'-AACCAATCCAACCAAAACAA-3'. The PCR conditions used were the same as those described previously [30]. The PCR products were cloned into the pGEM-T easy vector (Promega, Madison, WI), amplified, and sequenced in our institutional DNA sequencing facility with T7 primer.

### ChIP assay

The ChIP assay was performed with a kit from Millipore (Billerica, MA), according to the manufacturer's protocol, with two clones (#5D11 and 8E11) of anti-BPDE antibodies (Trevigen, Gaithersburg, MD).

### Protein extraction and Western blotting

Total cellular protein was isolated for Western blotting as previously described [17-19]. The antibodies used were anti-c-Jun/AP-1, anti-DNMT3A (Santa Cruz Biotechnology, Santa Cruz, CA), anti-COX-2 (DB Transduction Laboratories, Lexington, KY), anti-phosphorylated-Erk1/2, anti-DNMT3B (Cell Signaling Technology, Beverly, MA), and anti-β-actin antibody (Sigma).

### PCR analysis of the RAR-β_2 _gene promoter

The DNA-protein complex from the ChIP assay was then subjected to PCR analysis. The primers used for the RAR-β_2 _gene promoter were 5'-TCATTTGAAGGTTAGCAGCCCGGGTA-3' and 5'-GGAGGCAAATGGCATAGAAA-3', which generated a 502-bp PCR product after 35 cycles.

### DNMT3A shRNA and transient gene transfection

DNMT3A shRNAs were purchased from OriGene Technologies (Rockville, MD). They were used for knocking down DNMT3A expression using Lipofectamine 2000 (Invitrogen) and treated with 0.5 μg/mL of puromycin for 48 h. The total cellular protein from these cells was subjected to Western blotting analysis of DNMT3A expression, and RNA from duplicate experiments was subjected to RT-PCR analysis of RAR-β_2 _expression as previously described [36].

5-Aza, 5-aza-2'-deoxycytidine; MSP, methylation-specific polymerase chain reaction; BPDE, benzo [*a*]pyrene diolepoxide; ChIP, chromatin immunoprecipitation; CpG, cytosine-phosphate-guanine; DNMT-3A, DNA (cytosine-5-)-methyltransferase 3 alpha; RT-PCR, reverse transcription-polymerase chain reaction; RAR-beta2, retinoic acid receptor-β_2_.

## Competing interests

The authors declare that they have no competing interests.

## Authors' contributions

XCX developed the experimental design and prepared the manuscript for publication. FY performed the experiments. Both authors read and approved the final version of the manuscript.
